# Transitional cell carcinoma with extension of the renal vein and IVC tumor thrombus: report of three cases and literature review

**DOI:** 10.1186/s12957-016-1041-z

**Published:** 2016-12-28

**Authors:** Mingyang Li, An Shi, Wen Kong, Jin Zhang, Yonghui Chen, Jiwei Huang, Yiran Huang

**Affiliations:** Department of Urology, Renji Hospital, School of Medicine, Shanghai Jiao Tong University, 1630 Dongfang Road, Shanghai, 200127 China

**Keywords:** Renal pelvic tumor, Transitional cell carcinoma, Tumor thrombus

## Abstract

**Background:**

Transitional cell carcinoma (TCC) originating from the renal pelvis with a venous tumor thrombus is a rare entity. However, clinicians should be aware of it because of its high malignancy and poor prognosis.

**Case presentation:**

Here, we report three cases of pathologically confirmed TCC originating from the renal pelvis with extension into the renal vein or inferior vena cava (IVC). Of these patients, two are males and one is female (58~73 years old). Their main symptom is flank pain; besides, gross hematuria and weight loss is observed in one of the patients. Computed tomography (CT) scan of the first patient revealed multiple space-occupying lesions in the left renal pelvis and left medium and lower ureter with a tumor thrombus in the left renal vein. CT scan of the second patient revealed a right renal mass and extension into the IVC. Abdominal magnetic resonance imaging (MRI) of the third patient showed a soft tissue mass in the region of the left renal sinus, and the signal of the soft tissue was observed in the left renal vein. The preoperative diagnoses of the first and third patient were TCC, while the second patient was renal cell carcinoma (RCC). Two patients with the preoperative diagnosis of TCC underwent laparoscopic radical nephroureterectomy with thrombectomy, and the other patient underwent radical nephrectomy with thrombectomy. The surgeries were successful. Although two of our patients underwent chemotherapy and radiotherapy, they died 2 and 19 months after the surgery, respectively. The other patient refused any adjuvant therapy and died 3 months after the operation.

**Conclusions:**

Compared to the extension of RCC to the renal vein or IVC, extension of TCC to the renal vein or IVC is rare. TCC with a venous tumor thrombus is often misdiagnosed as RCC. However, a correct preoperative or intraoperative diagnosis is of great importance to decide surgical strategy. Laparoscopic radical nephroureterectomy with thrombectomy may be a safe and feasible operative method in treatment of TCC with a renal vein thrombus. The prognosis of such cases is poor even if chemotherapy and radiotherapy are scheduled.

## Background

Renal cell carcinoma (RCC) and adenocarcinoma account for about 85% of all solid neoplasms of the kidney [1], while transitional cell carcinoma (TCC) accounts for 10 to 15% of all primary renal malignancies [2]. Although a venous tumor thrombus is not uncommon in RCC, TCC with a venous tumor thrombus is rare. So far, it has been described in 26 English-language literatures [1–26], and it was often misdiagnosed as RCC preoperatively in the literature. Usually, the operative method in treatment of TCC with a venous tumor thrombus is open radical nephroureterectomy with thrombectomy. Herein, we presented three cases of pathologically confirmed TCC originating from the renal pelvis with extension into the renal vein or inferior vena cava (IVC), and two of our patients underwent laparoscopic surgeries.

## Case presentation

### Case 1

A 73-year-old man who had been suffering from left flank pain for 20 days was referred to our outpatient clinic for evaluation and treatment. No significant past medical or surgical history was elicited. No palpable abdominal mass was detected during the physical examination. His laboratory evaluation revealed mild renal function impairment (white blood cell count [WBC] 9.41 × 10^9^/L, hemoglobin [Hb] 144 g/L, percentage of neutrophil cell 78.4%, platelet count [PLT] 234 × 10^9^/L, lactate dehydrogenase [LDH] 528 U/L, blood urea nitrogen [BUN] 6.70 mmol/L, creatinine [Cr] 124.3 μmol/L, C-reactive protein [CRP] 28.0 mg/L, and erythrocyte sedimentation rate [ESR] 37). Urinalysis showed microscopic hematuria and proteinuria.

A plain abdominal X-ray revealed a suspected left renal mass. A three-phase computed tomography (CT) urography revealed left hydronephrosis and multiple space-occupying lesions in the left renal pelvis and left medium and lower ureter. The left renal parenchyma was totally invaded and multiple enlargements of lymph nodes were seen in the aortocaval area. Thus, a TCC was highly suspected. Furthermore, an abdominal CT angiography showed a tumor thrombus in the left renal vein. No obvious metastasis was observed on chest CT except for some tiny nodules.

As the preoperational diagnosis was TCC from the left renal pelvis with the left renal vein thrombus, left laparoscopic radical nephroureterectomy with thrombectomy and regional lymphadenectomy were performed. No neoplasm was observed in intraoperative cystoscopy examination. The left kidney was enlarged, solid, and adherent to perirenal fat. The left medium and lower ureter was dilated, solid, and adherent to iliac vessels. The thrombus was gently milked back into the left kidney and removed along with the kidney.

Gross examination of the specimen showed necrosis in the left renal calyx and wall of the left medium and lower ureter. Postoperative histopathological examination revealed high-grade TCC (16 × 9 × 10 cm) (according to the 2004 WHO classification), which infiltrated parenchyma and perirenal fat diffusely and extended into the renal vein. Postoperative histopathology also showed multiple para-aortic lymphatic metastases, of which the biggest one was 6 cm (pT4N3M0). No microscopic lymphovascular invasion was observed. The margin of the ureter was negative.

No significant event was observed during the perioperative period. The patient underwent chemotherapy in another center, but the regimen was not recorded. The patient died 2 months after the operation.

### Case 2

A 58-year-old man who had been suffering from right flank pain for 1 year complained of aggravation of the pain for 1 month. Furthermore, there was a weight loss of 10 kg of the patient over the past 1 year. Neither palpable abdominal mass nor renal percussive pain was detected during the physical examination. The result of laboratory examination was almost normal except for the increase of CRP level (WBC 6.24 × 10^9^/L, Hb 118 g/L, PLT 96 × 10^9^/L, BUN 7.10 mmol/L, Cr 104.1 μmol/L, and CRP 11.50 mg/L). Urinalysis showed microscopic hematuria and proteinuria.

An abdominal CT showed a right renal mass and extension into the IVC. Nothing was abnormal on chest CT. The same finding was also showed on a PET/CT. According to the radiographic imaging findings, we suggested the diagnosis to be right RCC without metastasis. Then, the patient underwent open right radical nephrectomy and thrombectomy without regional lymphadenectomy. After ligation of the right renal artery, the contralateral renal vein, proximal and distal IVC were partially clamped to prevent the thrombus from falling off. The tumor thrombus was then completely removed. At surgery, we found severe adhesion of the right renal hilum to the IVC and an enlarged lymph node near the right renal pedicle. The operation was successful. No complications were observed during the perioperative period.

Postoperative histopathological examination revealed high-grade TCC (10 × 8 × 5.5 cm), which infiltrated parenchyma, with massive necrosis. The thrombus also consisted of high-grade TCC. The perirenal fat was negative of carcinoma cell (pT3N0M0), while the margin of ureter was positive. No microscopic lymphovascular invasion was observed. As for adjuvant therapy, the patient received radiation therapy for 1 month and chemotherapy with taxol and carboplatin for one cycle. The patient died of cancer 19 months after the surgery.

### Case 3

A 68-year-old woman presented to our outpatient clinic with asymptomatic gross hematuria and left flank pain for 1 month. The patient had a history of diabetes mellitus for 10 years and lacunar infarction for 1 year. The physical examination was normal. The results of routine laboratory examinations were remarkable for leukocytosis and mild anemia (WBC 12.66 × 10^9^/L, Hb 103 g/L, percentage of neutrophil cell 79.7%, PLT 177 × 10^9^/L, BUN 7.00 mmol/L, Cr 115.0 μmol/L, CRP 8.360 mg/L, ESR 30). Her urinary nuclear matrix protein 22 (NMP 22) was weakly positive.

**Fig. 1:**
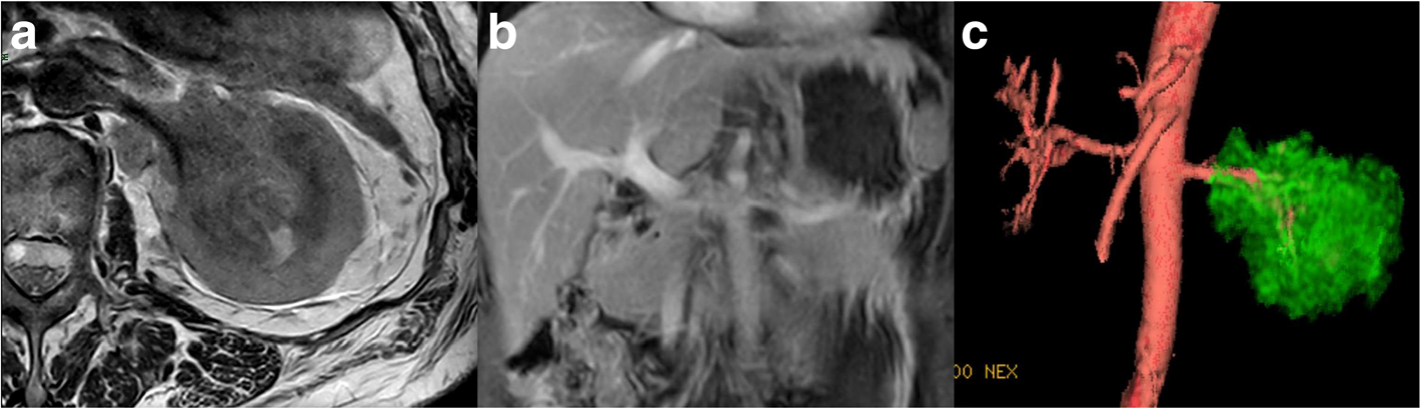
Radiological findings of renal pelvic TCC with renal vein thrombosis. **a** Case 3: axial MRI image showed a soft tissue mass in the region of the left renal sinus with parenchyma and perirenal fat infiltrated. The signal of soft tissue was also seen in the left renal vein. **b** Case 3: coronal MRI image showed renal vein thrombosis. **c** Case 3: MRI 3D reconstruction image showed a mass in the region of the left kidney

A renal contrast-enhanced ultrasonography revealed left hydronephrosis and a space-occupying lesion in the left renal pelvis, which was suggested to be renal pelvic carcinoma, with a thrombus in the left renal vein. Abdominal magnetic resonance imaging (MRI) showed a soft tissue mass in the region of the left renal sinus with parenchyma and perirenal fat infiltrated. The signal of soft tissue was also seen in the left renal vein (Fig. 1). Multiple retroperitoneal lymph node metastases were suspectable. No obvious distant metastasis was observed on chest CT or bone scanning. Thus, left invasive TCC with the renal vein tumor thrombus was strongly suspected preoperatively.

**Fig. 2:**
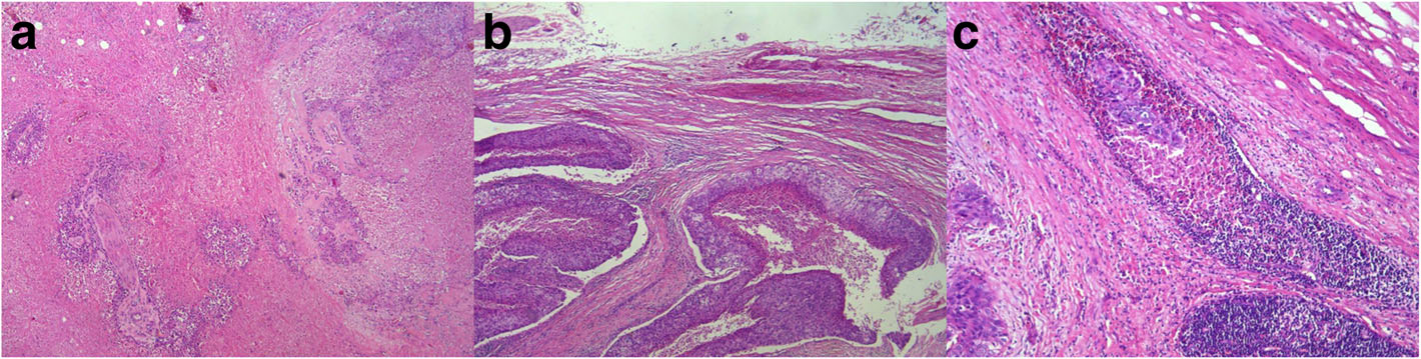
Pathological findings of renal pelvic TCC with renal vein tumor thrombosis. **a** Case 3: high-grade TCC with parenchyma diffusely infiltrated. **b** Case 3: tumor thrombus consisting of high-grade TCC cell. **c** Case 3: hilar lymph node positive of TCC cell

Then, the patient underwent left laparoscopic radical nephroureterectomy with thrombectomy and regional lymphadenectomy. At surgery, the left kidney was slightly adhesive to surrounding tissues. Enlarged lymph nodes were seen in the region of the renal hilum and abdominal aorta. Meanwhile, the left renal artery was severely adhesive to the lymph nodes nearby. Postoperative histopathological examination revealed high-grade TCC (6 × 5.5 × 3 cm), with parenchyma diffusely infiltrated (Fig. 2a). The renal vein thrombus was composed of the same carcinoma cell (Fig. 2b). The perirenal fat and margin of the ureter were negative of carcinoma cell, while a hilar lymph node was positive (pT3N1M0) (Fig. 2c). Microscopic lymphovascular invasion was observed near the left renal hilum. The patient refused any adjuvant therapy and died 3 months after the operation.

### Discussion

Compared to the extension of RCC to the renal vein or inferior vena cava, the incidence of which reported to be 4 to 36% [27], extension of TCC to the renal vein or inferior vena cava is relatively rare. Previous studies have analyzed the incidence of a venous tumor thrombus in renal TCC, and the result showed that the frequency of a venous tumor thrombus was 5 to 7% [5, 26]. To the best of our knowledge, it has been described in 26 English-language literatures (Table 1) [1–26]. Herein, we report three cases: one with a tumor thrombus in the IVC and another two with a tumor thrombus in the renal vein. The symptoms of our patients are flank pain, gross hematuria, weight loss, and lower extremity edema, while the patients who were without gross hematuria commonly have microscopic hematuria in their laboratory evaluations. Meanwhile, anemia and renal function impairment are common too. In the previous literature, in which symptoms are documented, flank pain (17/31) and gross hematuria (15/31) are the most common symptoms. Besides, weight loss (7/31), fever (4/31), incidental renal mass (4/31), fatigue (3/31), palpable abdominal mass (3/31), and lower extremity edema (3/31) are also common symptoms. Uncommon symptoms are appetite loss, lethargy, nonproductive cough, etc. The female-to-male ratio is 16/23, and the mean age is 63.3 (24~89) in the previous literature.

**Table 1:** Reported cases of TCC of the renal pelvis with a venous tumor thrombus

Reference	Year	Age	Sex	Symptoms	Cytology	Treatment	Pathology	Prognosis (months)	Stage	Renal vein or IVC	Preoperative diagnosis
Renert et al. [3]	1972	24	M	rt flank pain, gross hematuria	NA	N + VCR	TCC G2	NA	NA	IVC	RCC
Tarry et al. [4]	1982	63	F	rt flank pain, gross hematuria, fever	positive	NU + T + L	TCC G3	No evidence of disease (20)	NA	IVC	TCC
Hartman et al. [5]^a^	1983	72	M	Gross hematuria	NA	N + VCR	TCC G3	NA	T3N?Mx	IVC	TCC
		52	M	rt flank pain, weight loss, appetite loss, fatigue, nonproductive cough	NA	N + T	TCC	Died (3)	T4NxM1	IVC	RCC
		NA	4 M/2 F	NA	NA	NA	TCC	NA	T3	renal vein	NA
Jitsukawa et al. [6]	1985	71	M	Gross hematuria, palpable abdominal mass	positive	NU + T + L + R	TCC G3	NA	T3	IVC	TCC
Geiger et al. [7]	1986	73	F	bil flank pain, weight loss	NA	N + VCR + L	TCC G3	No evidence of disease (12)	NA	IVC	RCC
Chang and Ma [8]	1987	58	F	Fatigue, weight loss, UTI	positive	NU + T	TCC G3	Died (5)	NA	IVC	TCC
Goldfarb et al. [9]	1990	81	M	Gross hematuria, weight loss	NA	N + T + L + C	TCC G2	No evidence of disease (18)	NA	IVC	RCC
Novick et al. [10]	1990	NA	NA	NA	NA	N + T	TCC G2	No evidence of disease (28)	NA	IVC	NA
Concepcion et al. [11]	1991	65	F	lt flank pain, lethargy	NA	N + T + L	TCC G3	Died (1)	NA	IVC	RCC
Leo et al. [12]	1992	78	M	Gross hematuria, weight loss, fever	positive	NU + T + L	TCC G3	No evidence of disease (9)	NA	IVC	TCC
		56	F	rt flank pain, gross hematuria, fever	negative	NU + T	TCC G3	Died (2)	NA	IVC	TCC
		60	F	Lower back and abdominal pain	negative	biopsy	TCC G3	Died immediately	NA	IVC	RCC
Vleeming et al. [13]	1994	76	M	rt flank pain, gross hematuria	NA	N + T + L	TCC G3	Died (6)	NA	IVC	RCC
Williams et al. [2]	1996	75	M	Gross hematuria	negative	N + VCR	High-grade TCC	Died (10)	NA	IVC	RCC
Oba et al. [14]	1997	62	M	rt flank pain, gross hematuria, fever, weight loss	positive	NU + T + L + C	TCC G3 + SCC	Died (5)	T3N2M0	IVC	TCC
Tajima et al. [15]	1997	72	M	rt flank pain	negative	biopsy + C	TCC	Alive (12)	T3	IVC	RCC
Fujimoto et al. [16]	1997	64	F	NA	positive	N + T	TCC G2	NA	NA	IVC	RCC
Miyazato et al. [17]	2001	47	M	Incidental lt renal mass, bil leg edema	negative	N + VCR	TCC G3	Died (17)	T3	IVC	RCC
Juan et al. [18]	2003	50	F	rt flank pain, gross hematuria, palpable rt abdominal mass, bil leg edema	negative	N + T	High-grade TCC	Died (3 weeks)	T3	IVC	RCC
		72	F	Chills, fever, rt flank tenderness	positive	NA	TCC	Died (5)	T3	IVC	TCC
Mahmood et al. [19]	2004	60	F	Hematuria, rt flank pain	negative	N + T + C	TCC G3 + squamous differentiation	No evidence of disease (3)	T4N0M0	renal vein	RCC
		55	F	Hematuria, rt flank pain, palpable rt abdominal mass	negative	N + T + C	TCC G3	Died (2)	T3N2M0	IVC	RCC
Cerwinka et al. [20]	2009	NA	M	Hematuria	NA	N + T	High-grade TCC	NA	T3	IVC	RCC
		NA	NA	Hematuria	NA	NU + T	High-grade TCC	NA	T3	IVC	RCC
Tseng et al. [21]	2010	62	M	lt leg edema	positive	C	High-grade TCC	Alive (9)	NA	IVC	TCC
Young and Kunju [22]	2012	34	M	NA	NA	N	High-grade TCC	NA	T4	renal vein	TCC
Nam et al. [23]	2012	67	M	rt flank pain	positive	NU + VCR	TCC G3	No evidence of disease (9)	T4N1M0	IVC	TCC
Pirola et al. [24]^b^	2013	NA	NA	NA	NA	NU + T + C	TCC G3	Mean survival 14.25 months	T3 ~ 4 N + M+	IVC	TCC
Diaz et al. [1]	2014	61	M	Incidental rt renal mass, fatigue	NA	N + T + L + C	High-grade TCC, sarcomatoid differentiation	NA	T4N0M0	IVC	RCC
Wang et al. [25]	2014	79	F	NA	NA	N + T	High-grade TCC	No evidence of disease (24)	T3N0M0	IVC	NA
Huber et al. [26]	2014	77	M	Incidental renal mass	NA	N + T	TCC G2	Died (9)	T4N0M0	IVC	RCC
		47	M	Incidental renal mass	NA	N + T	TCC G3	Died (60)	T4N0M0	IVC	RCC
		66	F	Flank pain	NA	N + VCR	TCC G3	Died (6)	T4N1M1	IVC	RCC
		58	M	Flank pain, weight loss	NA	NU	TCC G3	Died (3)	T4N3M1	renal vein	TCC
		89	F	Flank pain, weight loss	NA	N	TCC G3	Died (13)	T4N0M0	renal vein	RCC
Present cases		73	M	lt flank pain,	NA	NU + T + L + C	High-grade TCC, necrosis	Died (2)	T4N3M0	renal vein	TCC
		58	M	rt flank pain, weight loss	NA	N + T + R + C	High-grade TCC, necrosis	Died (19)	T3N0M0	IVC	RCC
		68	F	Hematuria, lt flank pain	NA	NU + T + L	High-grade TCC	Died (3)	T3N1M0	renal vein	TCC

*N* nephrectomy, *NU* nephroureterectomy, *T* thrombectomy, *L* lymphadenectomy, *VCR* vena cava resection, *C* chemotherapy, *R* radiotherapy, *UTI* urinary tract infection, *rt* right, *lt* left, *bil* bilateral, *NA* not applicable

^a^Eight cases

^b^Four cases

Radiological examination is of vital importance to the diagnosis of renal neoplasm. As the TCC develops in a similar location with RCC and infiltrates renal parenchyma, differentiation of TCC from RCC becomes much more difficult. In previous literature, correct preoperative diagnosis of TCC was made in 43% of the cases (16/37). A previous study tried to prove that CT scan is accurate in distinguishing intrarenal TCC from centrally located RCC with six CT features: (1) the tumor is centered within the collecting system; (2) a focal filling defect appears in the pelvicalyceal system; (3) the maintenance of the reniform shape of the kidney is present; (4) the necrotic or cystic change is absent; (5) the tumor is of homogeneous enhancement; and (6) the tumor extends toward the ureteropelvic junction [28]. In addition, there are obvious attenuation differences between TCC and RCC in the corticomedullary and nephrographic phases using multiple small regions of interest (ROIs) on multiphase CT scans [29], but there is still debate as to whether CT scan is sufficient to guide the management. Some researchers proposed that cytology, retrograde pyelography, and biopsy are meaningful in differentiation diagnosis and should be performed along with CT scan [30]. Although differentiation between RCC and TCC with parenchyma infiltrated by radiological examination is difficult, CT, MRI, and contrast-enhanced ultrasonography are pretty helpful to find a tumor thrombus [14]. Among these imaging modalities, MRI can reveal the size and position of a thrombus more precisely. Although selective renal arteriography of TCC of the renal pelvis often shows a hypovascular area in the section of tumor infiltration, a renal TCC with IVC thrombus can be hypervascular on the contrary [15]. Taken together, combination of CT, pyelography, cytologic analysis of the urine, and biopsy will help urologists to differ renal pelvic invasive TCC from RCC.

In such cases, it is very important to make a correct preoperative diagnosis of the tumor type because the operative methods of TCC and RCC are different. Nephroureterectomy is suggested for treatment of TCC while nephrectomy for RCC [18]. A frozen section during surgery will help to differentiate TCC from RCC [1]. As for our case 2, we believe that we would perform a complete ureterectomy following the right radical nephrectomy and thrombectomy to avoid a positive ureter margin after an intraoperative frozen section.

In previous reports of TCC with a venous thrombus, nephrectomy with thrombectomy (or vena cava resection) and nephroureterectomy with thrombectomy (or vena cava resection) are main operative strategies (57 and 34%, respectively). Simple nephrectomy without thrombectomy and simple nephroureterectomy without thrombectomy are infrequent (6 and 3%, respectively). Wang et al. [25] first reported pure laparoscopic nephrectomy with thrombectomy in 2013. We performed laparoscopic surgery in two of our patients. As far as we know, we were the first ones to report laparoscopic nephroureterectomy with thrombectomy in treatment of TCC with extension into the renal vein. The operations were successful without perioperative complications. They were discharged at the seventh and eighth day after surgery, respectively. And, their renal function remained stable 1 month after surgery with creatinine being 72.6 and 124.7 μmol/L, respectively. Thus, we consider that laparoscopic radical nephroureterectomy with thrombectomy may be a safe and feasible operative method in treatment of TCC with a renal vein thrombus.

Patients with a renal vein or vena cava thrombus originating from TCC of the renal pelvis have a poor prognosis [17, 24]. Half of the patients who had documented follow-up in previous reports died of cancer within 10 months after surgery. The present cases died 2, 19, and 3 months after surgery, respectively, which agree with the former finding. The cases published so far, including our cases, in which stages or tumor grades were recorded, all had late stages (T3 ~ 4) and high grades, which may be relevant to the poor prognosis. In such cases, chemotherapy is the main adjuvant therapy (13/14), and the mean survival time of the patients who underwent chemotherapy (including our patients) is 10.6 months. The patient who only underwent radiotherapy had no follow-up data. One of our patients received radiotherapy along with chemotherapy (taxol and carboplatin for one cycle) and died 19 months after the surgery. Adjuvant therapy may be effective but its role remains unclear due to the limits of a few cases.

## Conclusions

TCC originating from the renal pelvis with a venous tumor thrombus is rare. A correct preoperative or intraoperative diagnosis is of great importance to decide surgical strategy. Intraoperative frozen section is recommended in cases suspectable for TCC. Laparoscopic radical nephroureterectomy with thrombectomy may be a safe and feasible operative method in treatment of TCC with a renal vein thrombus. The prognosis of such cases is poor even if chemotherapy and radiotherapy are scheduled.

## Abbreviations

BUN: Blood urea nitrogen
Cr: Creatinine
CRP: C-reactive protein
CT: Computed tomography
ESR: Erythrocyte sedimentation rate
Hb: Hemoglobin
IVC: Inferior vena cava
LDH: Lactate dehydrogenase
MRI: Magnetic resonance imaging
PET: Positron emission tomography
PLT: Platelet count
RCC: Renal cell carcinoma
TCC: Transitional cell carcinoma
WBC: White blood cell count.
